# Wood-Derived Hydrogels as a Platform for Drug-Release Systems

**DOI:** 10.1021/acssuschemeng.0c08022

**Published:** 2021-01-22

**Authors:** Mario Culebras, Anthony Barrett, Mahboubeh Pishnamazi, Gavin Michael Walker, Maurice N. Collins

**Affiliations:** † Stokes Laboratories, School of Engineering, Bernal Institute, University of Limerick, Plassy Technological Park, Limerick V94 T9PX, Ireland; ‡ Department of Chemical Sciences, Bernal Institute, Synthesis and Solid State Pharmaceutical Centre (SSPC), University of Limerick, Plassy Technological Park, Limerick V94 T9PX, Ireland; § Health Research Institute, University of Limerick, Plassy Technological Park, Limerick V94 T9PX, Ireland

**Keywords:** lignin, cross-linking, cellulose, rheology, drug release

## Abstract

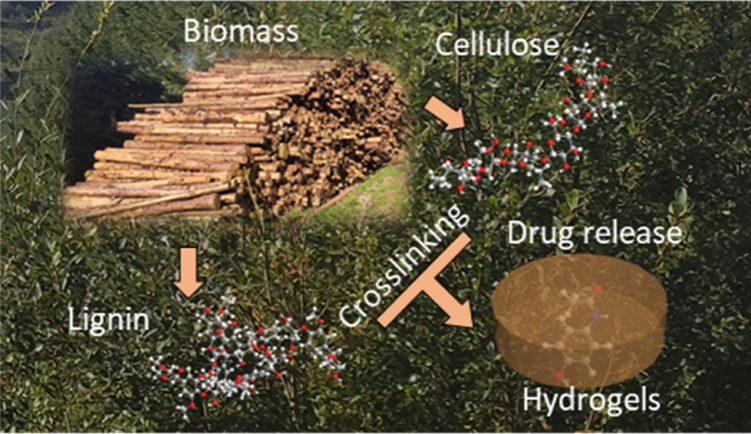

Wood (cellulose and lignin)-based hydrogels were successfully produced as platforms for drug-release systems. Viscoelastic and cross-linking behaviors of precursor solutions were tuned to produce highly porous hydrogel architectures via freeze-drying. Pore sizes in the range of 100–160 μm were obtained. Varying lignin molecular structure played a key role in tailoring swelling and mechanical performance of these gels with organosolv-type lignin showing optimum properties due to its propensity for intermolecular cross-linking, achieving a compressive modulus around 11 kPa. Paracetamol was selected as a standard drug for release tests and its release rate was improved with the presence of lignin (50% more compared to pure cellulose hydrogels). This was attributed to a reduction in molecular interactions between paracetamol and cellulose. These results highlight the potential for the valorization of lignin as a platform for drug-release systems.

## Abstract 2

Lignocellulosic biomass waste is a sustainable resource that can be valorized as novel drug-release platforms.

## Introduction

Controlled release systems allow tuning of drug dosage to specific rates, this keeps the drug concentration at an effective therapeutic level, thereby maximizing its effect within the body. In addition to controlled release, targeted release increases the efficiency of a drug as it allows delivery to a specific site in the body which requires the therapeutic.^1−4^ Consequently, novel platforms for controlled drug release are an area of interest in pharmaceutical science gaining considerable attention within the research community. Hydrogels are showing enormous potential due to their advantageous properties such as tunable time-dependent swelling behavior, biocompatibility, tunable mechanical behavior, and their ease of chemical modification.^5−8^ Hydrogels are typically defined as three-dimensional, hydrophilic, polymer networks capable of absorbing large amounts of water or biological fluids. Synthetic polymers such as poly(hydroxyalky1 methacrylates),^9,10^ poly(acrylamide),^11,12^ poly(methacrylamide), and poly(*N*-vinyl-2-pyrrolidone)^13,14^ have been extensively used in the preparation of hydrogels. Also, natural polymers such as alginate,^15,16^ hyaluronic acid,^17−19^ gelatin,^20,21^ and chitosan^22^ are widely used to make hydrogels for a variety of biomedical applications. However, there is a growing global awareness around sustainability of materials and its associated implications such as global warming and greenhouse emissions. It has therefore become important to produce new materials in more efficient ways from sustainable and renewable sources.^23^ Therefore, the use of undervalorized waste is crucial for the future sustainable development of society in order to avoid waste accumulation and to promote the circular use of resources. For example, agricultural and forestry lignocellulose waste represents more than 2 billion tons annually.^24^ Thus, lignocellulosic biomass represents the most abundant and biorenewable biomass on earth showing enormous potential for the production of new materials. Lignocellulose biomass consists of three main components: cellulose, hemicellulose, and lignin. The source of biomass determines the composition of each of these constituents.^23,25,26^ Cellulose is the most abundant and is considered a high-value product due to its application in the paper, textile, and biomedical sectors.^25,27−29^ However, lignin, the most abundant aromatic polymer in nature, is underutilized and is considered a nonvalorized waste. More than 50 million tons of lignin are available each year, produced as a byproduct from the paper and pulp industry. 98% is not isolated from black liquor and is burned on site for low-value energy purposes. There have only been a few studies on the usage of lignin for high-value applications, for example, the production of carbon-based materials such as carbon fibers for composites or nanostructured anode batteries.^30−36^ Uniquely, for a natural polymer, lignin is a natural source of phenolics and its branched molecular structure can be functionalized to produce tailored hydrogels for varying applications.

Looking at this scenario, this work focuses on the development of wood-derived hydrogels composed of cellulose/lignin blends to produce a new hydrogel platform for targeted and controlled drug-delivery platforms. In addition, structure/property relationships of these hydrogels are mapped as a function of composition and lignin type to provide a comprehensive understanding of these systems. This work allows new opportunities for lignin valorization in the pharmaceutical field.

## Experimental Section

### Materials

Alcell organosolv hardwood lignin (TCA) with an MW_n_ of 4000 g/mol (*T*_g_ = 100 °C, phenolic hydroxyl content 2.4 mmol/g) and lignosulfonate (TCS) (MW_n_ of 7000 g/mol, sulfonated content, 1.5–2.5 mmol/g) were obtained from Tecnaro GmbH (Germany). Sodium hydroxide (NaOH) pellets of 98% purity were purchased from AppliChem GmbH (Germany). Urea powder was purchased from Sigma-Aldrich GmbH (Germany). Microcrystalline cellulose (MCC SANAQ 101) was obtained from Pharmatrans Sanaq AG (Switzerland). Epichlorohydrin (ECH) 99% was obtained from Sigma-Aldrich GmbH (Germany). Paracetamol (4-acetamidophenol) was purchased from Phion Chemicals (United Kingdom).

### Preparation of Cellulose/Lignin Hydrogels

Initially, a NaOH/urea water solution was prepared adding 5.25 g of NaOH and 3.5 g of urea to 87.75 mL of water. The solution was mixed for 30 min until the NaOH pellets and urea were fully dispersed. The solution was then filtered with filter paper to remove any impurities. Then, 3.5 g (3.5 wt %) of MCC was added to form a total mass of 100 g including NaOH, urea, MCC, and water, this was magnetically stirred for 1 h and the solution was stored at −20 °C overnight. The frozen solid was left to thaw out at room temperature before being stirred to obtain a colorless and transparent solution. The organosolv solutions (TCA) were produced by mixing 3.5 g (3.5 % wt) of TCA with 5.25 g of NaOH solution to form a total mass of 100 g. This solution was mixed overnight to ensure the dissolution of the TCA in the solution. Lignosulfonate solutions (TCS) were prepared as mentioned above. Lignin/cellulose solutions were prepared by combining both solutions with a total mass of 10 g. Table 1 summarizes the prepared compositions.

**Table 1:** Summary of Hydrogel Formulations

sample	cellulose solution (g)	TCA solution (g)	TCS solution (g)	ECH (mL)
cellulose 0.5 mL	10.0			0.5
cellulose 1 mL	10.0			1.0
cellulose 5 mL	10.0			5.0
TCA 95:05	9.5	0.5		1.0
TCA 90:10	9.0	1.0		1.0
TCA 75:25	7.5	2.5		1.0
TCS 95:05	9.5		0.5	1.0
TCS 90:10	9.0		1.0	1.0
TCS 75:25	7.5		2.5	1.0

All solutions were cross-linked with ECH over 12 h at room temperature to form stable hydrogels. Hydrogels were rinsed several times with deionized water to remove excess NaOH and urea. The structural analysis of the samples carried out by Fourier transform infrared spectroscopy (FTIR) is shown in the Supporting Information (Figures S1 and S2).

### Freeze-Dried Hydrogels

The gels were freeze-dried using a Eurotherm freeze-dryer under the following conditions: initial freezing at −30 °C for 8 h at atmospheric pressure, primary drying at −10 °C for 16 h at 0.1 mBar and secondary drying: 20 °C for 2 h at 0.1 mBar. Prior to undergoing the freeze-drying process, the gels were stored at −80 °C overnight.

### Characterization

Compression tests were carried out using a Tinius Olsen compression tester (United Kingdom) at a compression rate of 0.5 mm/min equipped with a 1 kN load cell. Tests were replicated three times.

Rheological testing was carried out on a TA Instruments Discovery HR-2 rheometer (USA) using 25 mm stainless-steel disposable plates with a loading gap at 25 mm. Flow sweep and strain sweep tests were performed at room temperature on the non-cross-linked samples at a frequency of 1.6 Hz. The viscosities were measured as a function of shear rate from 0.5 to 500 s^–1^. Samples were subjected to frequency sweeps from 0.1 to 100 Hz keeping the strain at a constant value of 2%. Time sweep tests were carried out for each sample upon addition of 1 mL of ECH cross-linker to monitor the evolution of viscoelastic properties during the cross-linking reaction. Each time sweep lasted 2 h. All solutions were magnetically stirred for 10 min before undergoing testing.

Morphological analysis was carried out by scanning electron microscopy (SEM) in a Hitachi TM-1000 (United Kingdom). Freeze-dried samples were fractured and mounted in the SEM sample holder. Prior to analysis samples were gold-sputtered. The accelerating voltage during SEM observation was 15 kV.

Swelling tests were conducted at 37 °C in a PolyScience water bath (USA) in phosphate-buffered saline (PBS) solution. Prior to testing, gels were dried overnight in a Gallenkamp vacuum oven at 600 Bar with the temperature set at 50 °C. Prior to immersing the gels in PBS, their dry weight was recorded using a Sartorius balance. After the gels were placed in the water bath, their weight was measured periodically over the course of 26 h. Before each weighing, the gels were dried on blotting paper to remove any surface water from the gels. The % swelling of the gels was calculated as follows
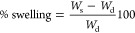
1where *W*_s_ is the weight of the sample at each time point and *W*_d_ is the dry weight of the sample.

Gels underwent cargo loading in paracetamol solutions with a concentration of 10 mg/mL as described in previous studies.^37^ After drug loading, the gels were placed in baskets in individual chambers of a 900 mL solution in a Pharma Test dissolution machine. The baskets were rotated at a constant speed of 50 rpm. Aliquots of the drug-release solutions were measured over time using UV–vis spectroscopy (Agilent Technologies Cary 60 UV–vis spectrophotometer, USA). This analysis was performed to determine the paracetamol percentage present in the solution (calibration curve shown in Figure S4). The duration of each test was 7 h. Data are presented as mean ± standard deviation (s.d.) and analyzed using one-way analysis of variance (ANOVA). *P*-values < 0.05 were considered significant.

## Results and Discussion

**Figure 1:**
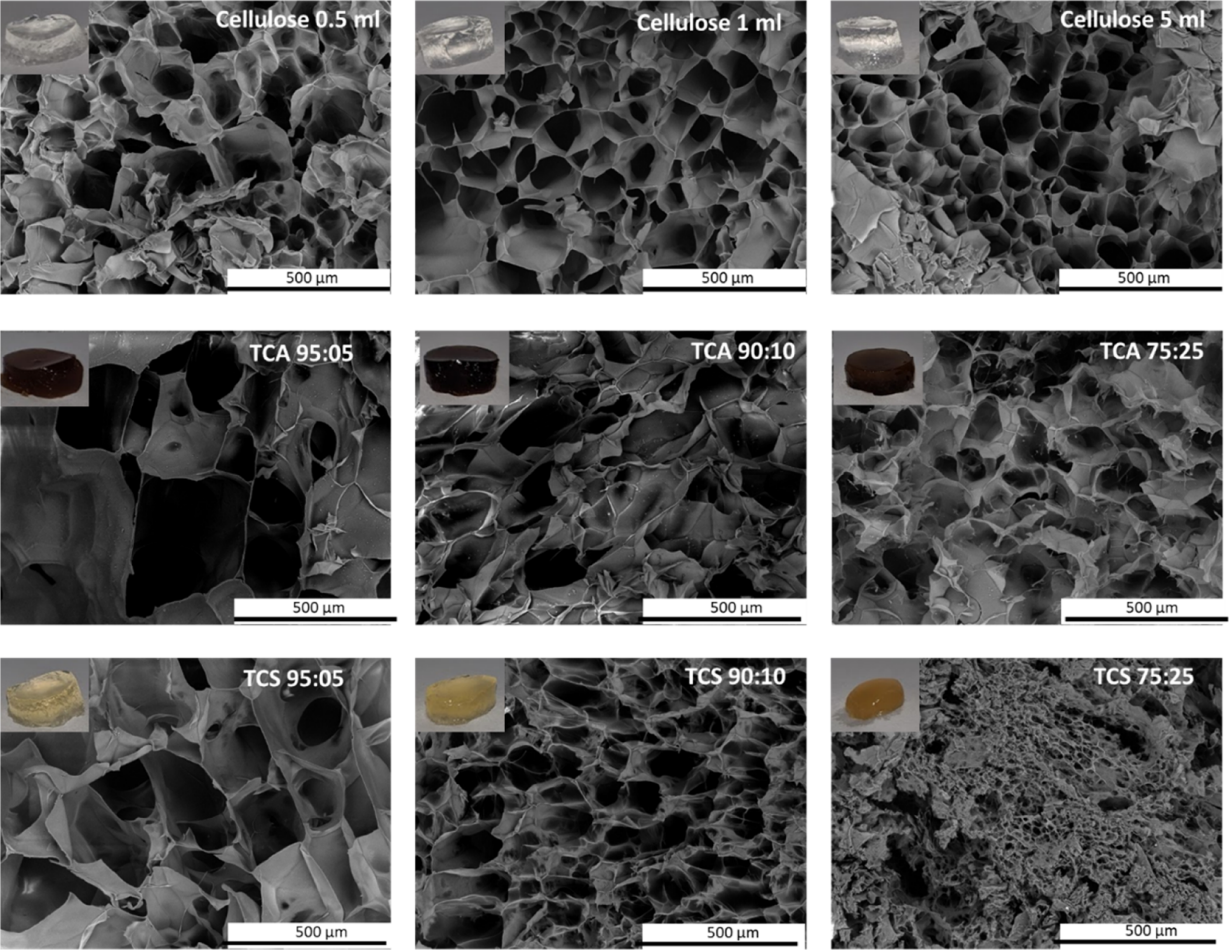
SEM images and pictures of the cellulose and cellulose/lignin hydrogels prepared.

**Figure 2:**
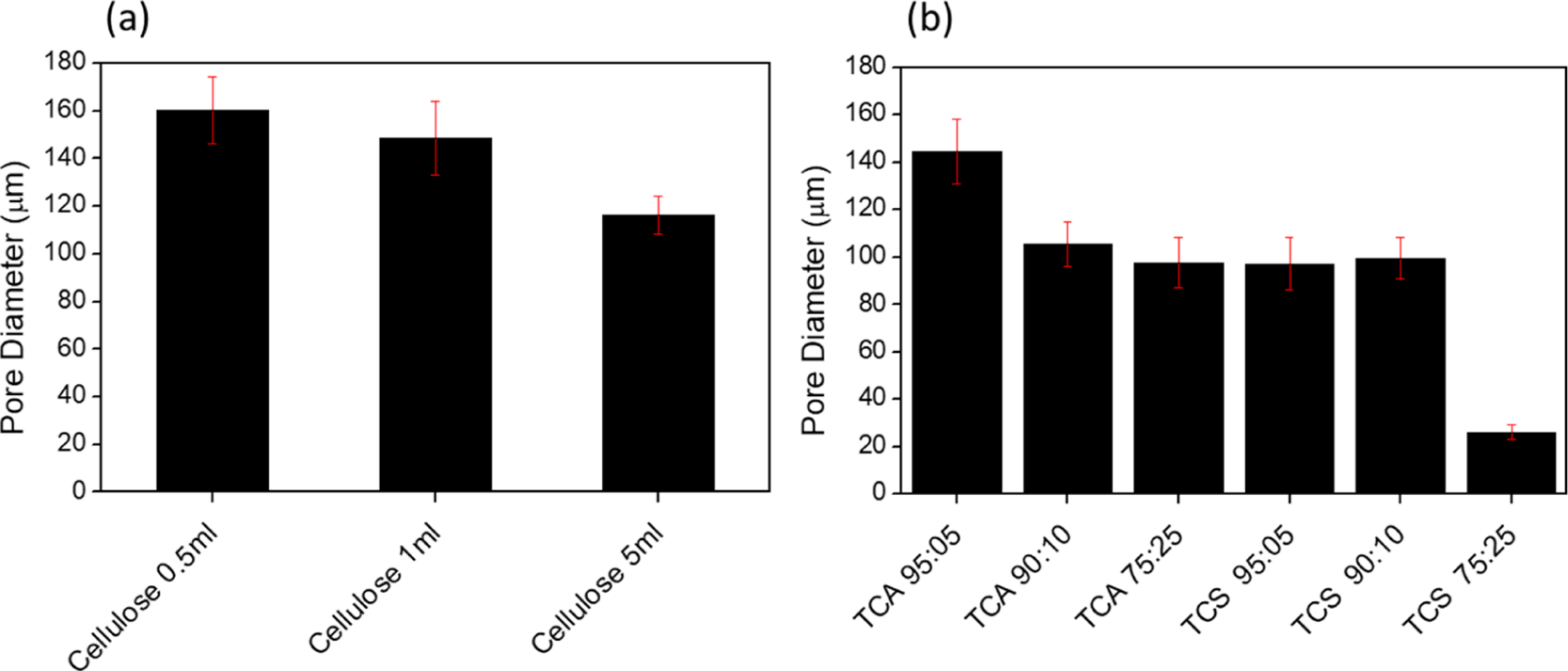
Pore size measurements of (a) cellulose cross-linked with: 5, 1, and 5 mL of ECH and (b) cellulose/lignin hydrogels cross-linked with 1 mL of ECH.

The optimum amount of ECH to cross-link the cellulose hydrogels was found to be in the range of 0.5 mL (5.5 % wt) to 1 mL (10.5 % wt). The sample prepared with 5 mL of cross-linker showed clear phase separation due to excess ECH, and samples containing 0.2 mL were not robust enough for molding. Figure 1 shows SEM images of freeze-dried cellulose and cellulose/lignin hydrogels. All hydrogels displayed a typical porous structure.^18,38^ For cellulose hydrogels, pore size decreased as a function of the amount of cross-linker, as shown in Figure 2a.

For lignin-based hydrogels, the pore morphology is similar to the pure cellulose hydrogels. However, the morphological evolution of TCA and TCS differed as a function of lignin content in the hydrogel. Taking organosolv lignin as an example, the porous structure remained similar at the three different ratios of lignin to cellulose (95:5, 90:10, and 75:25), although higher values were obtained for the cellulose/TCA ratio 95:05. For Figure 2b, TCA 95:05 hydrogels display the largest pore size of lignin-based gels being only slightly smaller than pores on the pure cellulose hydrogels. For TCA hydrogels, generally pore sizes decreased as lignin content increased. TCS 95:05 and TCS 90:10 hydrogels display similar pore sizes to TCA 75:25, while a TCS 75:25 hydrogels were approximately 30% smaller than 90:10 and 95:05 TCS hydrogels.

**Figure 3:**
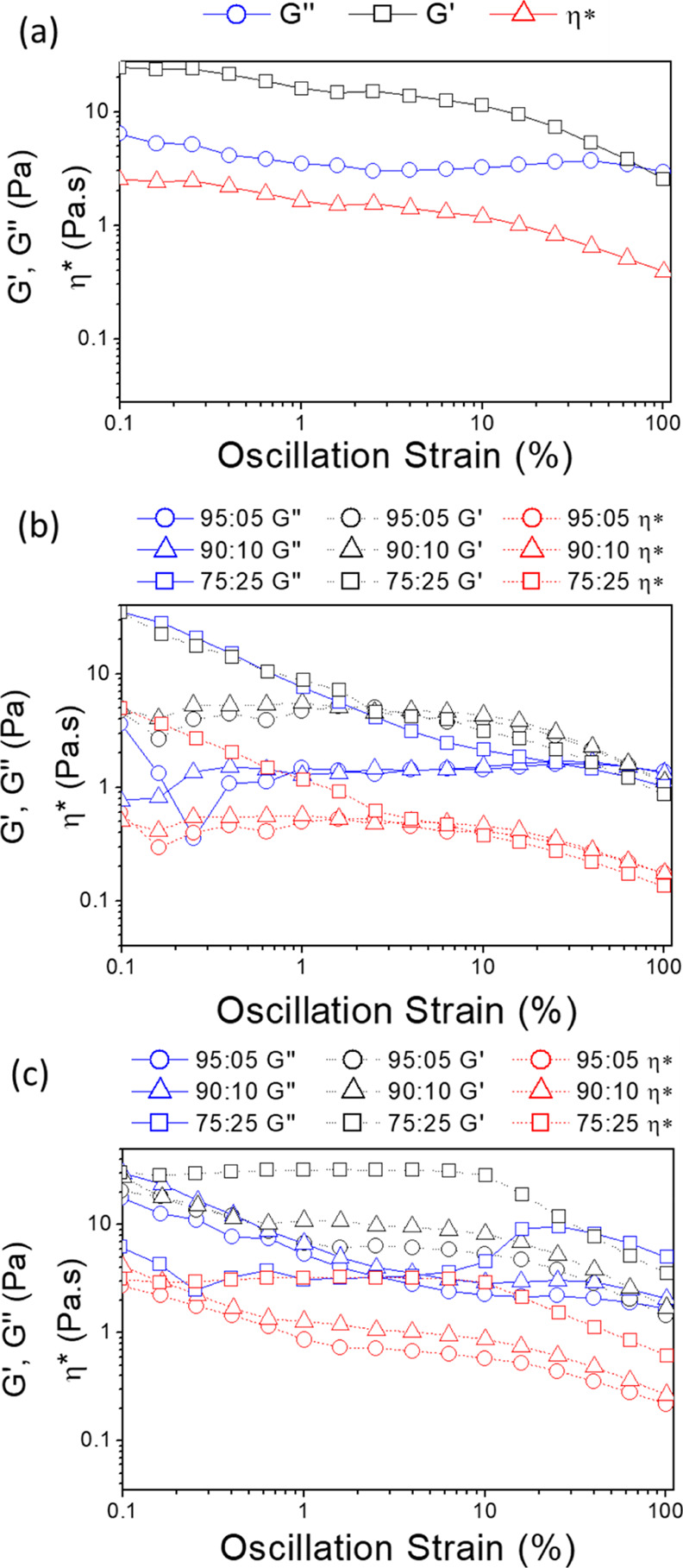
Viscosity (red line), storage (black line), and loss moduli (blue line) as a function of the oscillation strain of (a) cellulose, (b) TCA/cellulose, and (c) TCS/cellulose-based hydrogels.

The viscoelastic behavior of the hydrogel solutions was determined using rheology. Strain sweeps were carried out in order to determine the linear viscoelastic region of the solutions. Figure 3 illustrates the storage modulus, loss modulus, and complex viscosity as the solution underwent an oscillating strain. Cellulose and cellulose/lignin solutions with ratios 90:05 and 90:10 show a linear viscoelastic region until 10% oscillation strain where the storage and loss moduli are independent of the applied shear strain, *G*′ is higher than *G*″, which indicates a solid- or gel-like behavior. However, the viscosity, storage, and loss moduli begin to decrease at values higher than 10% of oscillation strain indicating a breakdown or disruption of the elastic network formed by the cellulosic polymer chains due to their intermolecular interactions. For cellulose/TCA 75:25, a lack of a linear viscoelastic region is explained by the presence of a higher amount of organosolv lignin with its branched chains disrupting the cellulose network due to intermolecular interactions between both polymers.

**Figure 4:**
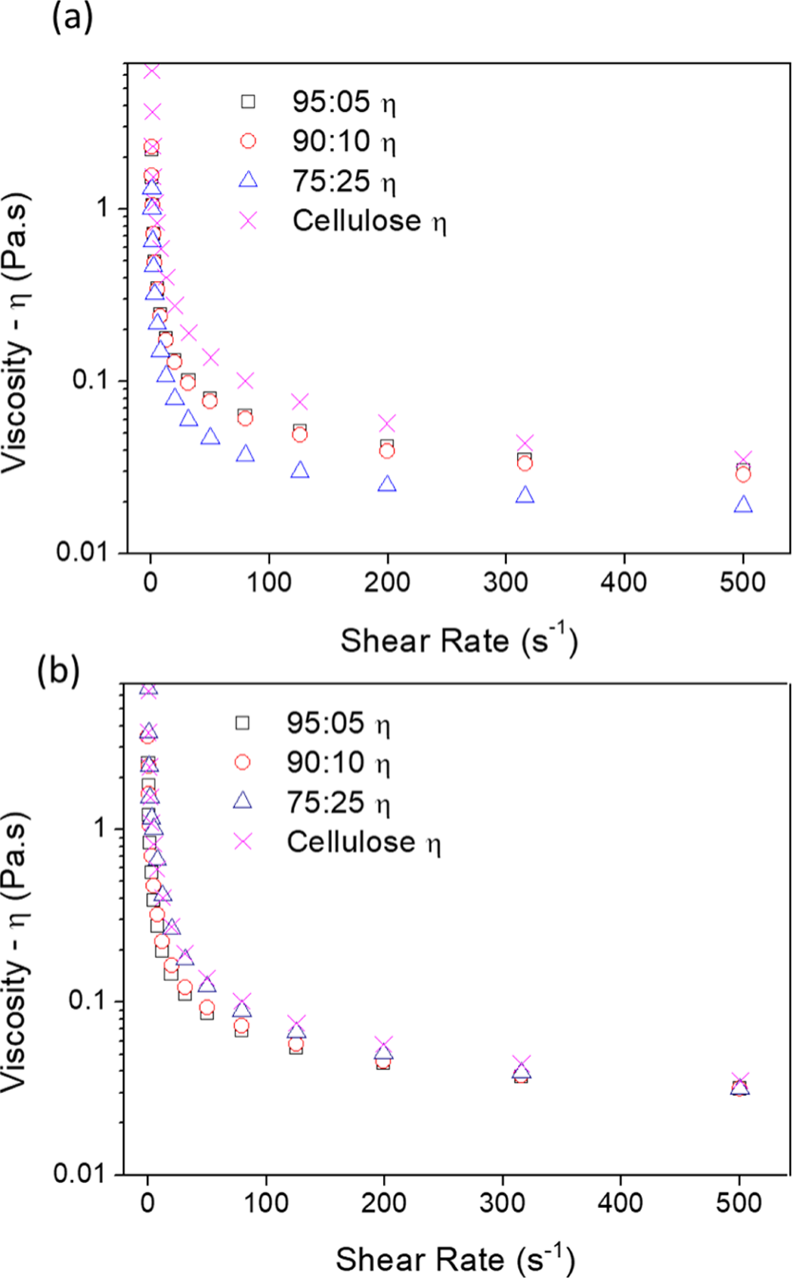
Viscosity as a function of the shear rate for (a) TCA/cellulose and (b) TCS/cellulose-based hydrogels.

Figure 4 shows the shear viscosity for TCA/cellulose and TCS/cellulose hydrogel solutions. Pure cellulose solutions show higher shear viscosities than lignin/cellulose solutions due to H-bond interactions between cellulose polymer chains creating a relatively strong network.^27,28^ However, when TCA is added to the cellulose solution, the shear viscosity decreases as a function of the lignin content (see Figure 4a). This may be attributed to the presence of phenolic groups in TCA that are susceptible to H-bonding with cellulose, thereby disturbing the cellulose network with the knock on effect of decreasing solution viscosity.^38^ In contrast, for TCS/cellulose solutions, the viscosities were similar compared to the pure cellulose solutions indicating that the cellulose network is not influenced by the presence of the TCS. This is attributed to the molecular structure of TCS which contains sulfonate functional groups that are less inclined to interact with cellulose.

**Figure 5:**
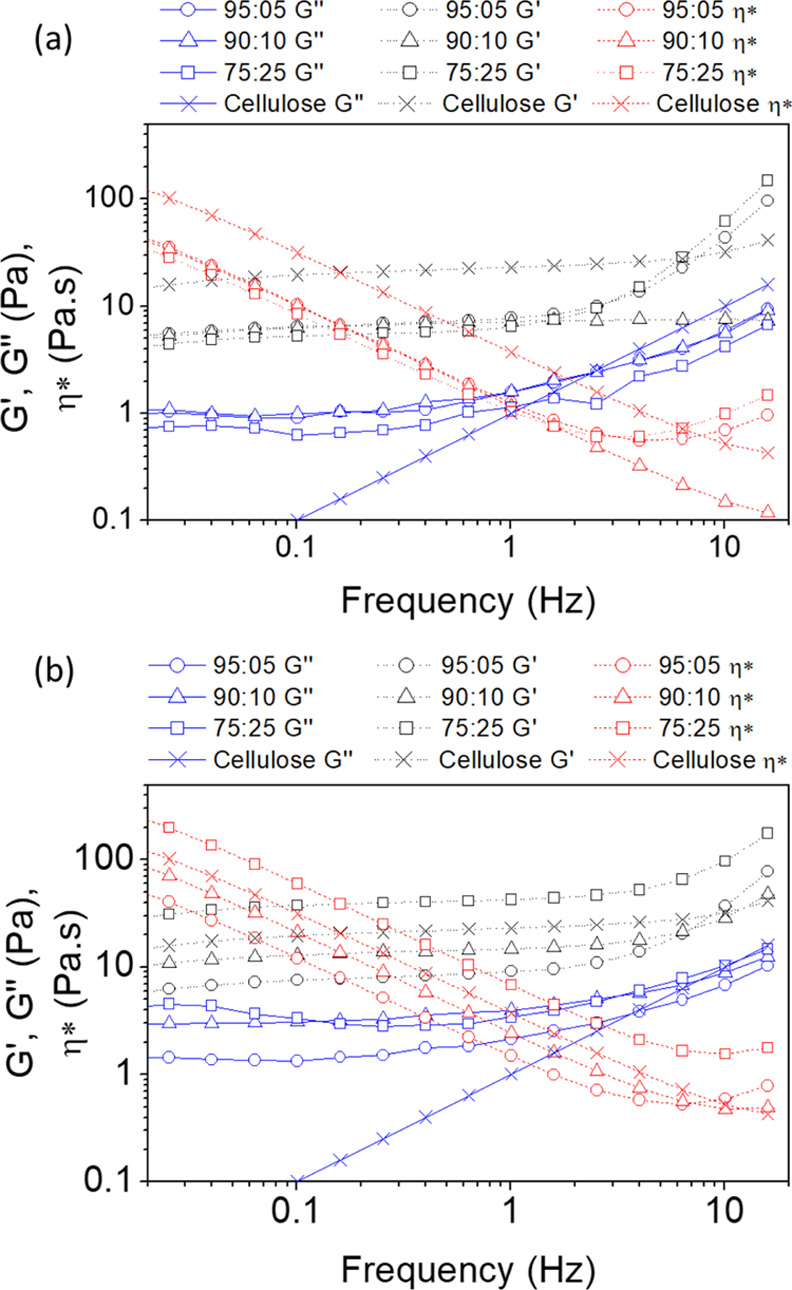
Viscosity, storage, and loss moduli as a function of the frequency of (a) TCA/cellulose and (b) TCS/cellulose hydrogels.

**Figure 6:**
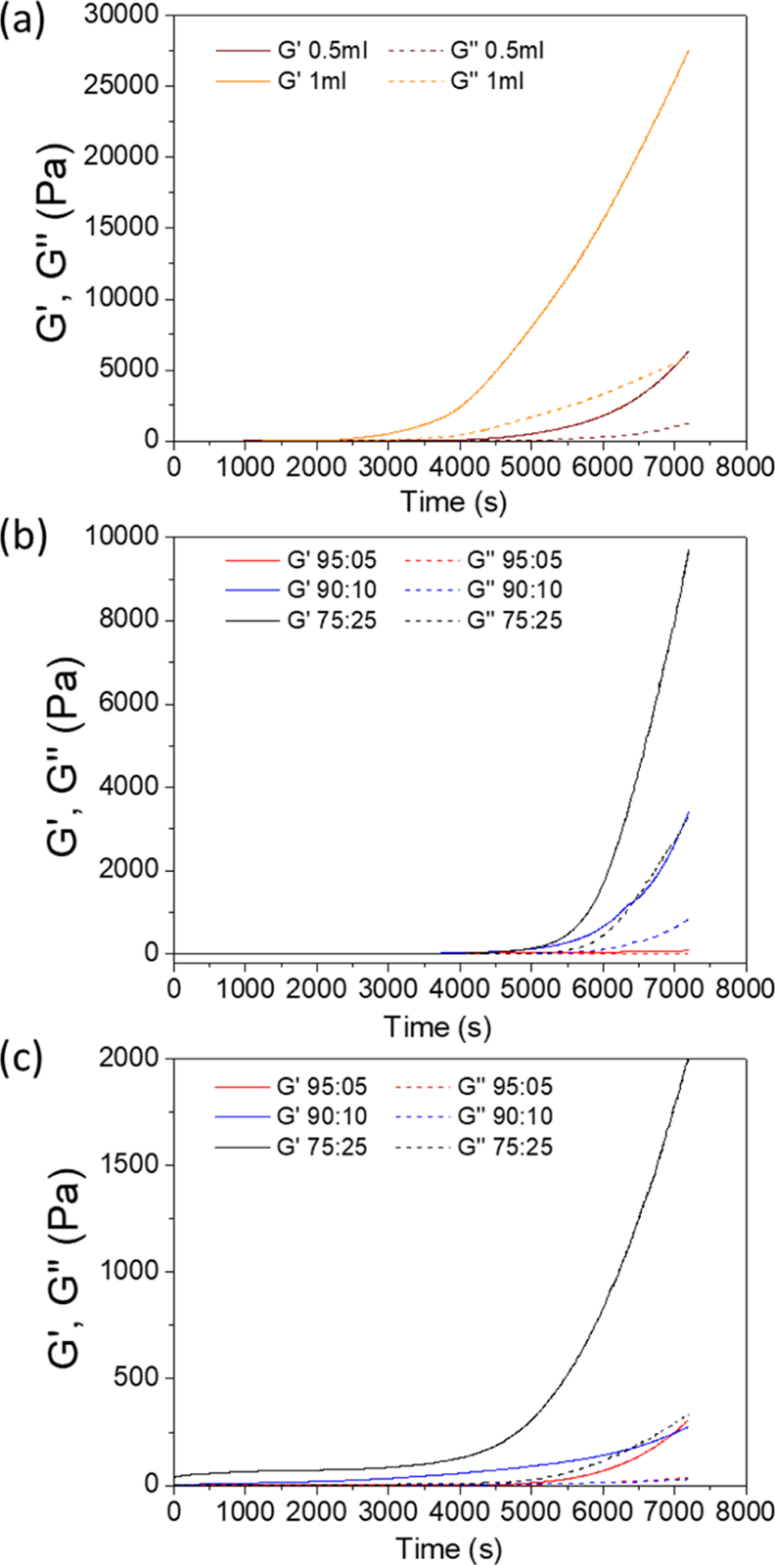
Storage and loss modules as a function of the time during cross-linking with ECH of (a) cellulose, (b) TCA/cellulose, and (c) TCS/cellulose-based hydrogels.

Figure 5 shows the storage, loss modulus, and the viscosity of cellulose/lignin solutions as a function of frequency. The storage modulus was higher than the loss modulus for all samples indicating an elastic behavior. The cross-linking process of the cellulose/lignin hydrogels was monitored by time sweep rheological measurements, as shown in Figure 6. For cellulose solutions (Figure 6a), upon addition of ECH, the storage and loss moduli increase indicating that the cross-linking process has initiated. Samples prepared with 1 mL of ECH display higher storage moduli compared to samples cross-linked with 0.5 mL of ECH, indicating higher cross-linking efficiencies. Results obtained for the cellulose/TCA and cellulose/TCS samples show a similar trend. The storage modulus increases as a function of time and it is higher for samples with higher lignin ratios presumably this is attributed to the higher reactivity of the lignin phenolic groups with ECH compared to the hydroxyl groups of cellulose.

**Figure 7:**
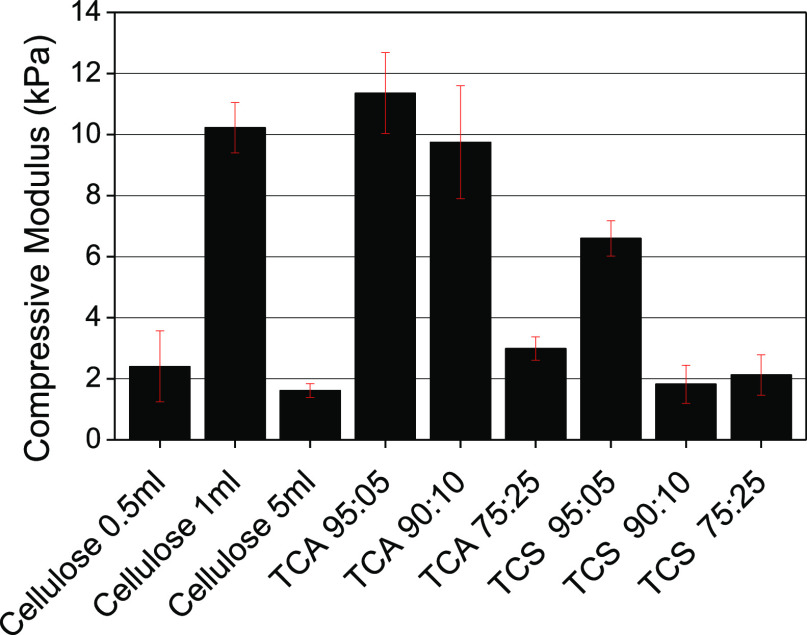
Compressive modulus obtained for the cellulose/lignin hydrogels calculated from the stress–strain curves.

Figure 7 illustrates the calculated compressive moduli from the stress–strain curves shown in Figure S3 for all hydrogels. These results show that by increasing the cross-link density, hydrogels exhibit greater mechanical stiffness up to a point. For example, the cellulose gel cross-linked with 5 mL of ECH exhibits the lowest compressive modulus of all the gels studied here. This is attributed to an excess of ECH that acts as a plasticizer reducing their mechanical properties. TCA 95:05 hydrogels exhibit the highest compressive moduli suggesting that lignin can reinforce the cellulose network at these levels via intermolecular interactions. However, at higher lignin ratios, the compression moduli decrease, indicating that the lignin chains are themselves being cross-linked, increasing the hydrophobic character of the hydrogel system, creating structural imperfections that negatively influence mechanical properties. In contrast, TCS-based hydrogels show lower values of compression moduli suggesting that the TCS molecular structure is not contributing to intermolecular cross-linking reactions between cellulose and TCS. Thus, TCS-based hydrogels exhibit a lower cross-linking degree and thereby lower mechanical performance compared to TCA-based hydrogels.

**Figure 8:**
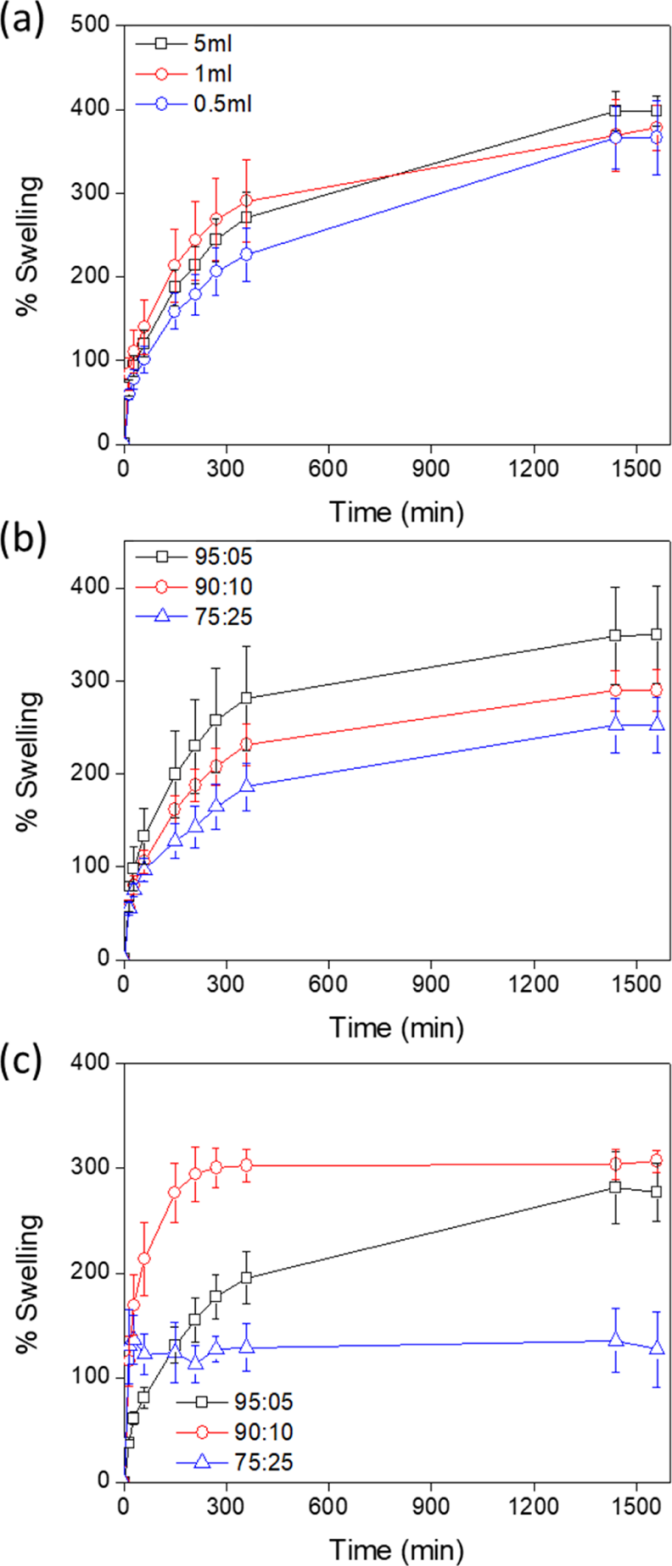
Swelling capacity of (a) cellulose, (b) cellulose/TCA, and (c) cellulose/TCS gels.

The swelling capacity of the hydrogels was measured in PBS and is shown in Figure 8. For pure cellulose hydrogels, relationships show that by increasing cross-linker content, the swelling capacity decreases slightly. Although results are similar, the swelling capacity of the cellulose solutions cross-linked with 5 mL of ECH decreased compared to equivalent samples cross-linked with 1 mL of ECH. This is attributed to increased cross-linking densities. For TCA samples, organosolv lignin content reduces swellability. This may be explained by the higher hydrophobic character of the organosolv lignin which reduces water imbibition in the hydrogel network. In contrast, for cellulose/TCS hydrogels, swelling increased. This arises due to the presence of ionic sulfonate groups in the TCS structure. However, for the samples with a cellulose/TCS ratio of 75:25, the swelling capacity was low as at these levels, TCS does not participate in intermolecular cross-linking with cellulose producing a weak hydrogel network as previous results have shown. The diffusion coefficient for each hydrogel was calculated using data obtained from the swelling tests assuming that the water uptake can be described by one-dimensional diffusion process; that is, radial diffusion can be neglected compared to axial, obeying Fick’s first law of diffusion.^39^ The diffusion coefficient, *D*, is determined using the following equation
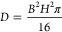
2where *H* is the thickness and *B* is determined from the slope of the plots *M*_*t*_/*M*_∞_ versus *t*^1/2^ (*M*_*t*_ is the swelling after a time *t* and *M*_∞_ is the swelling at equilibrium).

Table 2 shows the calculated diffusion coefficients for each of the gel variants. The results align with published data of diffusion coefficients calculated for similar hydrogels being in the 10^–4^ to 10^–5^ cm^2^ s^–1^ range.^40^ For lignin-based hydrogels, there is a decrease in the diffusion coefficient as TCA lignin content increases in the hydrogel which aligns with the idea that TCA reduces the water uptake in the hydrogels. The phenolic groups of lignin are responsible for the intramolecular cross-linking with cellulose chains; consequently, more aliphatic hydroxyl groups are generated due to the use of ECH as a cross-linker. Therefore, the number of ionizable groups (phenolic) decreases and consequently, the hydrophobicity of the hydrogel increases, thereby reducing water diffusion through the hydrogel. Conversely, increasing TCS content increases the diffusion coefficient due to the presence of sulfonate groups as the number of ionizable groups increases within the hydrogel network and therefore the diffusion coefficient increases accordingly.

**Table 2:** Diffusion Coefficients for Each Hydrogel System

sample	*B*^2^	*D* (cm^2^ s^–1^)
cellulose 0.5 mL	0.0347	(6.4 ± 0.2) × 10^–5^
cellulose 1 mL	0.045	(1.1 ± 0.4) × 10^–4^
cellulose 5 mL	0.037	(7.3 ± 0.2) × 10^–5^
TCA 95:05	0.046	(1.1 ± 0.2) × 10^–4^
TCA 90:10	0.0451	(1.1 ± 0.2) × 10^–4^
TCA 75:25	0.041	(8.9 ± 0.6) × 10^–5^
TCS 95:05	0.03871	(7.9 ± 0.1) × 10^–5^
TCS 90:10	0.0735	(2.9 ± 0.4) × 10^–4^

**Figure 9:**
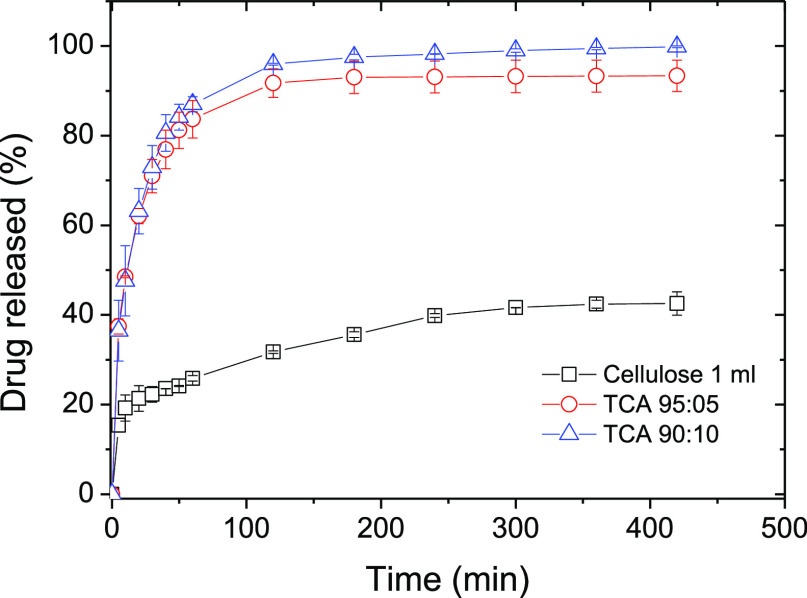
Paracetamol released from each hydrogel.

Paracetamol was selected as a standard drug for the release tests (Figure 9). The following hydrogels: cellulose 1 mL, TCA 95:05, and TCA 90:10 were selected for release studies as they displayed the most suitable characteristics as potential drug-release platforms. Platforms for drug-release studies were selected based on their mechanical performance, a key factor for the administration of these platforms as cylindrical shapes at implant sites. The addition of lignin increased drug release compared to pure cellulose hydrogels. This fact can be explained due to a higher affinity of paracetamol to cellulose compared to lignin. The molecular interactions by hydrogen bonds between cellulose chains and paracetamol molecules are responsible for its slow release, as demonstrated in our previous studies carried out using cellulose-based tablet formulations.^41^ The addition of lignin reduces the interaction between paracetamol and cellulose, increasing the diffusion of paracetamol from the lignin-containing hydrogels to the media. The results showed how the drug release can be controlled through the composition of the hydrogels since the amount of lignin present in the cellulose hydrogel significantly changed their release behavior. This offers a new opportunity for the valorization of lignin into high-value products in particular in the biomedical field as a platform for drug-release systems.

## Conclusions

Lignin (TCA and TCS) and cellulose represent an important class of abundant raw materials to produce high-end wood-derived products. An understanding of their structural, chemical, and mechanical behavior is crucial for the development of efficient and robust drug-release platforms. Herein, cellulose/lignin hydrogels were produced using two structurally different lignins, organosolv and sulfonated lignin. Resulting hydrogels display well-defined porous structures after freeze-drying. It was found that molecular interactions between components play an important role in their viscoelastic behavior with the organosolv-type lignin more susceptible to H-bond formation. In addition, varying lignin molecular structure drastically affects swelling capacity due to the introduction of hydrophobicity when utilizing organosolv lignin and this reduces water imbibition into the hydrogel network while the presence of ionic sulfonate groups in the TCS structure increases the swelling capacity of the hydrogels. Mechanical properties were highly dependent of the composition of the hydrogels. The cellulose/TCA ratio 95:05 shows the highest compression modulus indicating a reinforcement mechanism within the cellulose network via intermolecular interactions. Paracetamol was used as a model for release studies and release is improved in cellulose/TCA and this was attributed to the addition of lignin which reduces the paracetamol/cellulose interactions. Overall, these wood-based materials show promise as next-generation sustainable platforms for drug-release applications.

## Supporting Information Available

Fourier transform infrared (FTIR) analysis of cellulose and cellulose/lignin hydrogels, stress–strain curves of the compression test, and calibration of UV–vis analysis (PDF)

## Abbreviations

TCA: alcell organosolv hardwood lignin
TCS: lignosulfonate
MCC: microcrystalline cellulose
ECH: epichlorohydrin
SEM: scanning electron microscopy
PBS: phosphate-buffered saline
