# Supplementary material for: Wood-Derived Hydrogels as a Platform for Drug-Release Systems
Source: ACS Sustain Chem Eng. 2021 Jan 22;9(6):2515–22. doi: 10.1021/acssuschemeng.0c08022 (PMC8296679; doi:10.1021/acssuschemeng.0c08022)
Supplement: Supplementary file 1 — sc0c08022_si_001.pdf [file sc0c08022_si_001.pdf]

Supporting Information

Wood derived hydrogels as a platform for drug release systems

Mario Culebras[a], Anthony Barrett[a], Mahboubeh Pishnamazi[b] Gavin M. Walker[b] and Maurice N Collins[a,c]*

[a]Stokes Laboratories, School of Engineering, Bernal Institute, University of Limerick, Plassy Technological Park, Limerick, V94 T9PX, Ireland.

[b]Department of Chemical Sciences, Bernal Institute, Synthesis and Solid State Pharmaceutical Centre (SSPC), University of Limerick, Plassy Technological Park, Limerick, V94 T9PX, Ireland.

[c]Health Research Institute, University of Limerick, Plassy Technological Park, Limerick, V94 T9PX, Ireland.

Email: mario.culebrasrubio@ul.ie

Number of Pages: 5

Number of Figures: 4 (Figure S1-Figure S4)

FTIR Results

FTIR analysis for the freeze-dried hydrogels was carried out in a PerkinElmer Spectrum 100 FTIR spectrometer. Figure S1 shows the results obtained for the cellulose hydrogels.

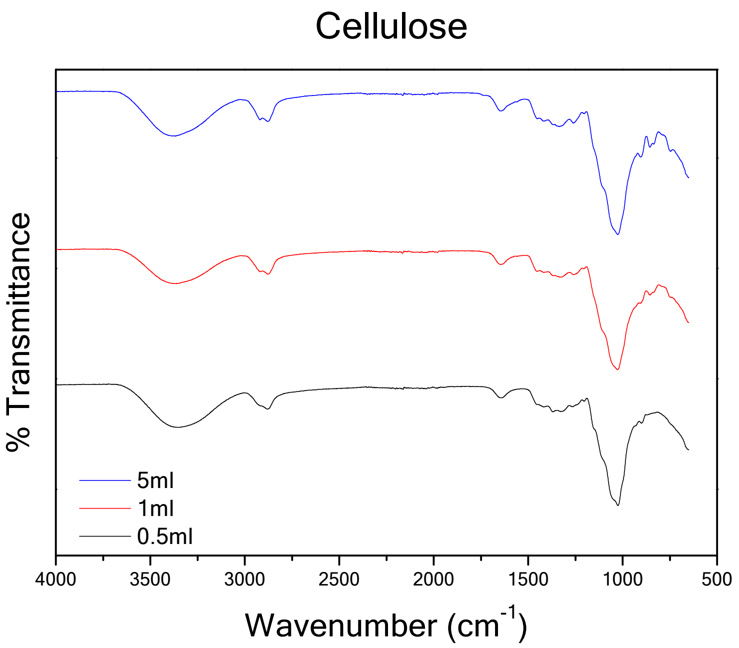

Figure S1. FTIR spectrum obtained for the cellulose hydrogels crosslinked with 0.5, 1 and 5 ml of Epichlorohydrin (ECH).

The band at 3390 cm^{-1} is related to the stretching of OH groups. The increased intensity of C-H stretching in the field of 2919 - 2878 cm^{-1} is seen with increased crosslinking density of ECH. In the range of 1638 - 1374 cm^{-1} appears deformation vibrations of CH_2 and CH groups located along with angular deformation vibrations of C-O-H. The bending vibrations of C-C groups are located in the band centered at 1025 cm^{-1}. Bending vibrations of C-H and ring vibration can be seen in the field 900-746 cm^{-1}.

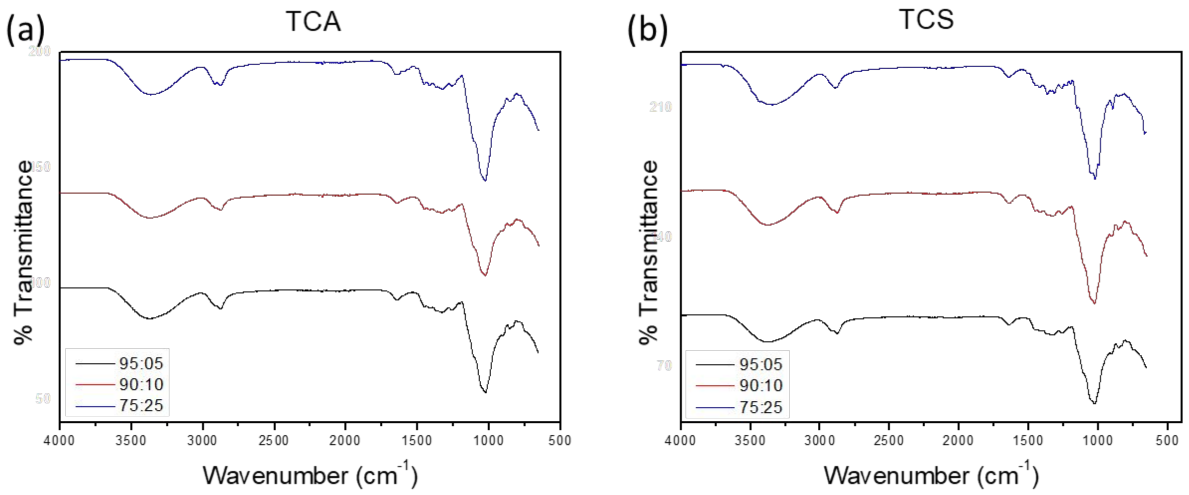

Figure S2. FTIR spectra for Cellulose/lignin hydrogels based on (a) TCA and (b) TCS formulations.

Figure S2 shows the FTIR analysis for Cellulose/lignin hydrogels. The spectra are very similar compared to the results obtained for pure cellulose samples indicating that lignin characteristic vibrations are masked by the cellulose bands.

Compression test results.

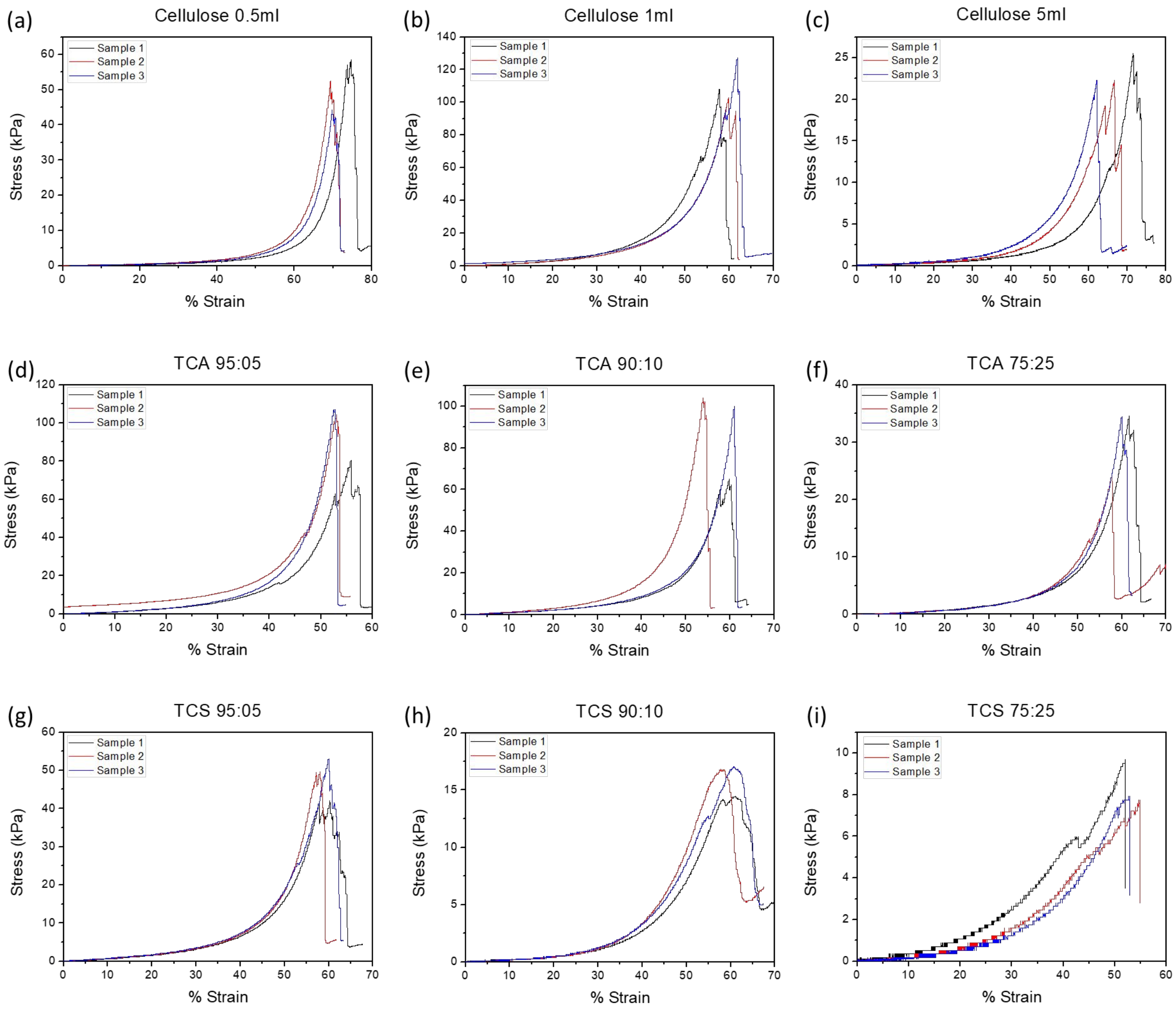

Figure S3. Stress-strain curves obtained for the different hydrogel variations.

Calibration

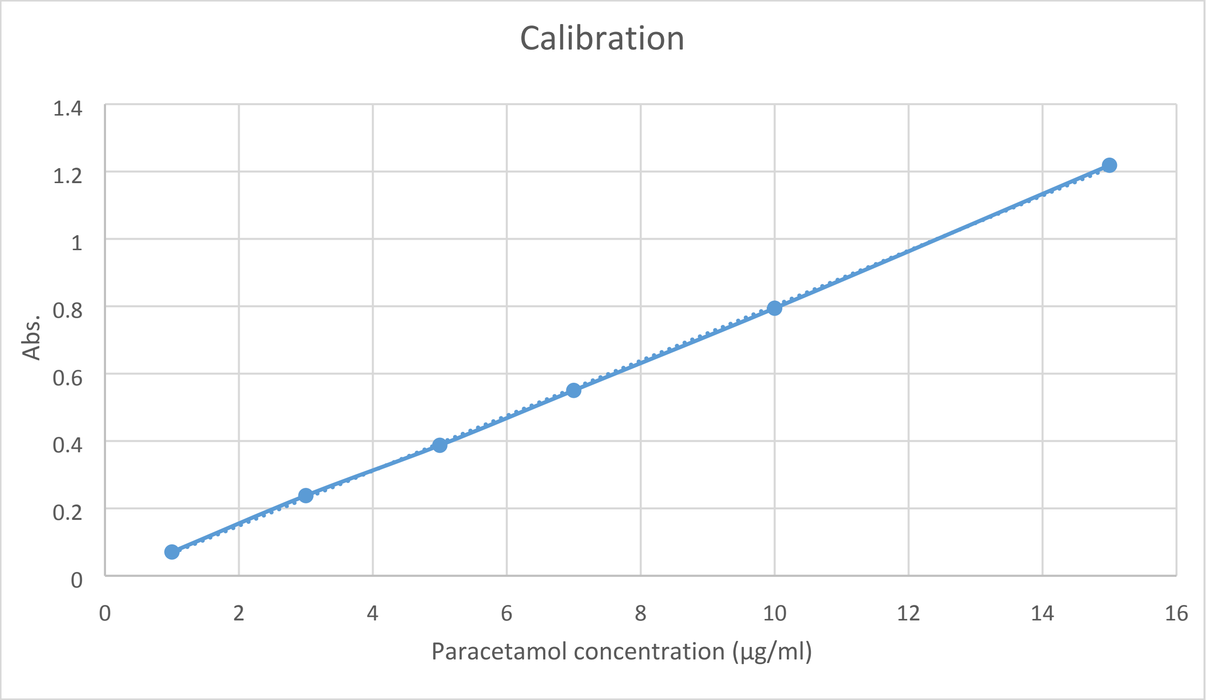

Figure S4. Paracetamol UV-Vis calibration curve.
